# The enhanced recovery after surgery (ERAS) protocol in elderly patients with acute cholecystitis: A retrospective study

**DOI:** 10.1097/MD.0000000000032942

**Published:** 2023-02-10

**Authors:** Tianyang Yu, Luwen Zhao, Hongtao Zhao, Hua Fu, Jian Li, Aijun Yu

**Affiliations:** a The First Department of General Surgery, Affiliated Hospital of Chengde Medical University, Chengde, Hebei Province, PR China; b The First Department of Gynecology, Affiliated Hospital of Chengde Medical University, Chengde, Hebei Province, PR China; c Surgical Operation Department, Affiliated Hospital of Chengde Medical University, Chengde, Hebei Province, PR China.

**Keywords:** acute cholecystitis, elderly patients, enhanced recovery after surgery protocol, laparoscopic cholecystectomy, perioperative

## Abstract

Enhanced recovery after surgery (ERAS) protocol is a perioperative management theory aimed at reducing the injury of surgical patients and accelerating postoperative recovery. It has been widely recognized and applied in elective surgery. This study aimed to evaluate the clinical value of the ERAS protocol during the perioperative period of laparoscopic cholecystectomy in elderly patients with acute cholecystitis. This study aimed to evaluate the clinical value of the ERAS protocol during the perioperative period of laparoscopic cholecystectomy in elderly patients with acute cholecystitis. We collected medical data from 126 elderly patients with acute cholecystitis from October 2018 to August 2021. Among the 126 patients, 70 were included in the ERAS group and 56 in the traditional group. We analyzed the clinical data and postoperative indicators of the 2 groups. No significant differences were observed regarding the general characteristics of the 2 groups (*P* > .05). The ERAS group had significantly earlier time to first flatus, time to first ambulation, and time to solid intake, compared with the traditional group (*P* < .001); additionally, the ERAS group had significantly shorter stay and gentler feeling of postoperative pain (*P* < .001). Furthermore, the ERAS group had significant incidences of lower postoperative lung (*P* = .029) and abdominal cavity infection (*P* = .025) compared to the traditional group. No significant difference was observed regarding the incidences of other postoperative complications between the 2 groups (*P* > .05). The ERAS protocol helps reduce elderly patients’ stress reactions and accelerate postoperative recovery. Thus, it is effective and beneficial to implement the ERAS protocol during the perioperative period of elderly patients with acute cholecystitis.

## 1. Introduction

The increased number of elderly individuals has led to an increased number of surgeries in different fields. Particularly, an increased number of emergency laparoscopic cholecystectomy (LC) has been observed due to the increased incidence of acute cholecystitis in elderly patients. Reducing the incidence of surgical complications and accelerating postoperative recovery is important for elderly patients. Henrik, a Danish surgeon, proposed the enhanced recovery after surgery (ERAS) protocol which aims to reduce patients’ trauma and stress injury and accelerate postoperative recovery. It consists of a series of measures performed during the perioperative period to reduce stress, response, postoperative complications, and hospital stay, and accelerate postoperative recovery.^[1–3]^ In China, the ERAS protocol has been widely used in clinical surgery. Currently, studies on the application of the ERAS protocol in LC have been reported; however, studies regarding its application to elderly patients with acute cholecystitis remain lacking.^[4]^ Therefore, this study aimed to observe the safety, efficacy, and feasibility of the ERAS protocol, compared with traditional perioperative management, in elderly patients who are undergoing emergency LC surgery for acute cholecystitis.

## 2. Material and methods

### 2.1. General information

This study retrospectively collected and reviewed the data of 126 elderly patients with acute cholecystitis who underwent LC surgery in the Department of Surgery of the Affiliated Hospital of Chengde Medical University from October 2018 to August 2021. Among the 126 patients, 70 were included in the ERAS group and 56 in the traditional group according to the use of ERAS measures. Diagnostic criteria for acute cholecystitis were determined according to the Tokyo Guidelines^[5]^: signs of local inflammation (Murphy positive); signs of mass/pain/tenderness in the right upper abdomen; signs of systemic inflammation (fever, elevated C-reactive protein, and elevated white blood cell count); and imaging findings of acute cholecystitis (ultrasound and computed tomography). Meeting one of the 3 items would confirm a diagnosis of acute cholecystitis. According to the World Health Organization’s demographics on aging, we adopted the standard of people >60 years old as the elderly.^[6]^ This study included patients with the following characteristics: age ≥ 60 years; diagnosed with acute cholecystitis according to the above-mentioned criteria; and the American Society of Anesthesiologists grade I to III. Patients with the following characteristics were excluded from the study: refused to provide consent for surgery; patients with comorbidities including severe hypertension, diabetes, and cardiovascular, hepatic, and renal dysfunction; comorbid bile duct calculi; onset time of >72 hours; and surgical conversion to laparotomy. The study was conducted in accordance with the Declaration of Helsinki and approved by the Ethics Committee of the Affiliated Hospital of Chengde Medical University.

### 2.2. Perioperative management

The ERAS group formulated perioperative management protocols based on Chinese expert consensus on ERAS in perioperative management (2016 edition).^[7]^ The perioperative management measures of the 2 groups are shown in Table 1.

**Table 1:** Perioperative management.

Measures	ERAS group	Traditional group
Preoperative period		
Education	The details of the surgery, the risks of intraoperative and postoperative complications and prophylaxis, the effects of anesthesia, and the aspects of post-anesthetic recovery were discussed with the patients prior to surgery. The patients received recommendations regarding physical activity and diet in the early postoperative period.	Traditional education of LC
Intraoperative period		
Temperature management	Body temperature was monitored, and a warm mattress was used	Operating room temperature was controlled
Fluid management	Target-directed fluid therapy was used	Conventional rehydration
Catheter	No catheter	Indwelling catheter
Postoperative period		
Postoperative analgesia	Multi-mode analgesia: ropivacaine incision infiltration + non-steroidal anti-inflammatory drugs	Analgesic pump automatic intravenous analgesia
Prevention and management of nausea and vomiting	Patients with high risk of postoperative nausea and vomiting were treated with 5-HT3 receptor antagonist and low-dose dexamethasone	No
Respiratory management	Patients were instructed to cough effectively, slap their chest and back, blow balloons, and inhale aerosolised glucocorticoids	No
Prevention of thrombosis	Bilateral lower limb barometric therapy was used to prevent deep venous thrombosis	No
Abdominal drainage tube removal time	One day postoperatively, bilirubin of drainage fluid was detected, and the abdominal drainage tube was removed after confirmation of the absence of bile leakage	The abdominal drainage tube was removed when there was no obvious liquid drainage
Other	Chewing gum was encouraged	

ERAS = enhanced recovery after surgery, LC = laparoscopic cholecystectomy.

### 2.3. Surgical method

The 2018 Tokyo Guidelines on the safety of LC for acute cholecystitis were followed to ensure patient safety.^[8]^ The operation must be performed within 72 hours of the onset of the illness. After tracheal intubation and general anesthesia induction, a transverse incision of 10 mm in length was made at the lower umbilical margin to establish CO_2_ pneumoperitoneum; additionally, the pressure was maintained at 12 mm Hg. A 10 mm incision was made below the xiphoid process, and a 5 mm incision was made 2 cm below the costal margin of the right midclavicular line. For difficult cases, a 5 mm incision was added to the anterior axillary line below the right costal margin. A trocar was inserted, the abdominal cavity was explored, and adhesions were separated. Subsequently, the common bile duct, cystic duct, and common liver duct were fully exposed. The cystic duct was clamped and separated 5 mm from the common bile duct. The cystic artery was bluntly separated and exposed in the inner side of the gallbladder triangle; subsequently, it was clamped by an absorbable clip and then cut off. The gallbladder was separated from the gallbladder bed by an electrocoagulation hook and placed in a specimen bag, and then removed from the abdominal cavity through the incision under the xiphoid process. An abdominal drainage tube was placed near the Winslow orifice.

### 2.4. Indicators

The visual analog pain scale (VAS) was used to score postoperative pain in both groups. The time to first flatus, time to first ambulation, time to solid intake, and hospital stay was recorded. Additionally, the incidence of surgical complications was recorded: unplanned reoperation or readmission within 30 days, death, biliary leakage, and infections of the urinary tract, incision site, abdomen, and lungs.

### 2.5. Statistical analysis

SPSS version 22.0 software (SPSS Inc., Chicago, IL) was used for statistical analysis. The measurement data were expressed as mean ± standard deviation (mean ± s). The counting data were expressed as values or rates (%). Bivariate comparison between groups was analyzed using 2 independent sample *T* tests, Pearson chi-square test and correction for continuity, and Fisher exact test where appropriate. Statistical significance was set at *P* < .05.

## 3. Results

### 3.1. Comparison of general information

General data of the 2 groups are shown in Table 2. No significant difference was observed regarding the general data between the 2 groups (*P* > .05).

**Table 2:** Patient demographics and clinical indicators.

	ERAS group	Traditional group	*t*/χ^2^	*P* value
Age, mean ± SD	67.67 ± 4.25	68.18 ± 5.08	0.610	.543
Sex (male/female)	34/36	23/33	0.706	.400
Onset time (h)	50.91 ± 6.38	49.59 ± 6.51	1.147	.254
ASA grade			0.167	.920
I	29	22		
II	28	22		
III	13	12		
Operative time (min), mean ± SD	58.09 ± 14.97	59.41 ± 13.70	0.513	.609
Pathological type (n)			0.307	.858
Acute simple cholecystitis	24	17		
Acute suppurative cholecystitis	31	18		
Acute gangrenous cholecystitis	15	11		

ASA = American Society of Anesthesiologists, ERAS = enhanced recovery after surgery, SD = standard deviation.

### 3.2. Postoperative recovery

The ERAS group had a significantly shorter time to first flatus, time to first ambulation, time to first feeding, and length of hospital stay compared to the traditional group (*P* < .05) (Table 3).

**Table 3:** Postoperative recovery of the 2 groups.

	ERAS group	Traditional group	*t*	*P* value
Time to first flatus (h)	24.04 ± 2.20	28.45 ± 3.95	6.005	<.001
Time to first ambulation (h)	7.44 ± 2.21	17.28 ± 6.86	11.300	<.001
Time to solid intake (d)	1.21 ± 0.42	1.87 ± 0.79	6.017	<.001
VAS score at 24-h after operation	2.99 ± 0.83	4.23 ± 0.95	7.862	<.001
Hospital stay (d)	3.17 ± 0.92	4.23 ± 1.29	5.381	<.001

ERAS = enhanced recovery after surgery, VAS = visual analog pain scale.

### 3.3. The incidence of postoperative complications

No significant difference was observed in the incidence of unplanned readmission, unplanned reoperation, biliary leakage, urinary tract infection, incision infection, and mortality between the 2 groups within 30 days postoperatively (*P* > .05). However, the ERAS group had significantly fewer cases of abdominal and pulmonary infection compared to the traditional group (*P* < .05) (Table 4).

**Table 4:** Incidence of surgical complications.

	ERAS group n (%)	Traditional group n (%)	*χ* ^2^	*P* value
Unplanned reoperation within 30 d	2 (2.86%)	4 (7.14%)	0.492	.483†
Unplanned readmission within 30 d	1 (1.43%)	3 (5.36%)	0.545	.460†
Death	0	0	–	–
Biliary leakage	2 (2.86%)	5 (8.93%)	1.182	.277†
Urinary tract infection	2 (2.86%)	4 (7.14%)	0.492	.483†
Incision infection	1 (1.43%)	1 (1.79%)	–	1.000‡
Abdominal infection	3 (4.29%)	9 (16.07%)	5.015	.025*
Pulmonary infection	14 (20%)	21 (37.5%)	4.749	.029*
Clavien–Dindo classification				
Grade I				
Incision infection	1 (1.43%)	1 (1.79%)	–	1.000‡
Biliary leakage	0	1 (1.79%)	–	.444‡
Grade II				
Urinary tract infection	2 (2.86%)	4 (7.14%)	0.492	.483†
Pulmonary infection	11 (15.7%)	12 (21.43%)	0.681	.409*
Pulmonary infection + abdominal infection	1 (1.43%)	5 (8.93%)	2.382	.123†
Grade IIIa				
Pulmonary infection + abdominal infection + biliary leakage	1 (1.43%)	2 (3.57%)	0.038	.845†
Grade IIIb				
Pulmonary infection + abdominal infection + biliary leakage	1 (1.43%)	2 (3.57%)	0.038	.845†
Grade ≥ IV	0	0	–	–

ERAS = enhanced recovery after surgery.

* Pearson chi-square test.

† Correction for continuity of Pearson chi-square test.

‡ Fisher exact test.

Patients with biliary leakage received follow-up treatment and recovered. A magnetic resonance cholangiopancreatography examination was performed in all patients with biliary leakage and no biliary duct injury was observed (Table 5).

**Table 5:** Treatment for patients with biliary leakage.

Patient	Group	Treatment	Treatment timing
1	ERAS group	Laparoscopic surgery for secondary diffuse peritonitis	Same admission
2	ERAS group	Ultrasound-guided puncture and drainage	Readmission within 30 d
3	Traditional group	Laparoscopic surgery for secondary diffuse peritonitis	Same admission
4	Traditional group	Laparoscopic surgery for secondary diffuse peritonitis	Same admission
5	Traditional group	Continuous drainage and removal on the sixth day after surgery	Same admission
6	Traditional group	Ultrasound-guided puncture and drainage were performed	Readmission within 30 d
7	Traditional group	Nasal bile duct drainage	Readmission within 30 d

ERAS = enhanced recovery after surgery.

### 3.4. Unplanned readmission within 30 days

In the ERAS group, one patient developed biliary leakage and underwent ultrasound-guided puncture and drainage after admission. Another patient developed fever and was treated with antibiotics after admission. In the traditional group, 2 patients developed biliary leakage; among them, 1 patient underwent ultrasound-guided puncture drainage, and the other underwent nasal bile duct drainage. Additionally, 2 patients developed fever and were treated with antibiotics after admission. All patients recovered.

## 4. Discussion

Since the 1990s, the application of laparoscopic technology in clinical practice has greatly promoted the development of minimally invasive surgery. Laparoscopic surgery is used for the treatment of various general surgical diseases, which can minimize the trauma and bleeding caused by previous open surgery. The advantages of laparoscopic technology include less trauma and bleeding, clear tissue anatomy, postoperative aesthetics, and quick recovery; additionally, it has been widely applied in all fields of surgery.^[9]^ The application of laparoscopy plays a great role in reducing patients’ stress and enhancing postoperative recovery, especially since the ERAS protocol was initiated. In elderly patients, LC has become the preferred strategy for acute cholecystitis.^[10]^

Changes in knowledge level, lifestyle, and social role have caused elderly people to have increased susceptibility to psychological and mental problems compared to young people. In the perioperative period, negative emotions of elderly patients such as anxiety and fear can adversely affect patients’ vital signs, such as heart rate and blood pressure, the therapeutic effect, and postoperative recovery. Simultaneously, many elderly patients with organ dysfunction will experience complications of various chronic diseases, such as hypertension, diabetes, malnutrition, anemia, and cardiopulmonary and renal insufficiency. Ensuring safety during the perioperative period of elderly patients who are indicated for emergency surgery is an important clinical issue.^[11]^ The ERAS protocol aims to provide patients with a more systematic, scientific, and rapid treatment and care through the integrated application of perioperative management; these factors achieve faster postoperative recovery and lower incidence of complications. The ERAS protocol requires the participation and close cooperation of surgeons, anesthesiologists, caregivers, and patients.

The ERAS protocol guidelines have been developed for use in different surgical areas, particularly in elective hepatobiliary and pancreatic surgery.^[12]^ However, guidelines for emergency surgery and the application for the surgical treatment of acute cholecystitis remain lacking.^[13]^ Therefore, this study made appropriate changes in perioperative management to adapt to the operation of acute cholecystitis. Particularly, LC is an emergency operation, which has a relatively short preoperative preparation time. Therefore, preoperative measures such as shortening fasting and drinking time, oral carbohydrate, smoking and alcohol prohibition, preoperative preventive application of analgesics, preoperative nutritional screening, and nutritional support were not implemented in the ERAS protocol. The ERAS protocol advocates that an abdominal drainage tube should not be placed intraoperatively. However, this study included patients with acute cholecystitis with heavy inflammatory exudation. Thus, the abdominal drainage tube was placed in both groups and was removed promptly in the ERAS group based on the condition of bilirubin in the drainage fluid.

In the ERAS group, the time to ambulation, the time to solid intake, and the time to first flatus were shorter than in the traditional group. In the ERAS group, chewing gum after surgery is a pseudo-feeding behavior, which stimulates various receptors in the mouth and reflexively causes enhanced gastrointestinal, pancreatic, hepatic, and cholecystic activities.^[14]^ A previous study showed that early postoperative oral intake of normal food or nutrient solution does not increase the rate of reoperation, complication, and mortality.^[15]^ In the ERAS group, a combined medication was used to prevent and inhibit postoperative nausea and vomiting, which can effectively avoid vomiting-induced aspiration and falling-down pneumonia, and reduce postoperative discomfort. Therefore, we encouraged patients in the ERAS group to be hydrated after they fully recover from anesthesia. Patients without abdominal distention were allowed oral food intake in the early stage without flatus and gradually transition from a liquid diet to a semi-liquid diet, soft diet, and normal diet. Early postoperative eating can effectively reduce the supply of venous fluid to avoid increasing the load on the heart. Additionally, early postoperative ambulation can avoid the risk of lung infection and venous thrombosis in patients who are long-term bedridden.

Compared with the traditional group, the ERAS group had lower postoperative VAS scores. Sufficient preoperative education enables patients to have a basic understanding of the disease diagnosis, surgical methods, perioperative management, and prognosis, which can relieve preoperative tension, reduce psychological stress, and allow psychological preparation for postoperative recovery. In the ERAS protocol, postoperative pain management adopts multi-mode analgesia, that is, combined applications of opioids, non-steroidal anti-inflammatory drugs, local incision infiltration anesthesia, intraspinal analgesia, and nerve block. Among them, in this study, incision local infiltration anesthesia and oral non-steroidal anti-inflammatory drugs were adopted, which can effectively relieve postoperative pain and avoid adverse reactions caused by strong opioids such as gastrointestinal peristalsis inhibition, nausea, and vomiting. Therefore, the ERAS group had significantly lower VAS scores, postoperative pain, nausea, and vomiting, compared to the traditional group.

Kamarajah’s meta-analysis found that advanced age is synonymous with increased major complications.^[16]^ Postoperative complication rates of LC ranged from 5.7% to 38.3% in previously published literature.^[17]^ In Fukami’s study, 16.9% of elderly patients with acute cholecystitis developed postoperative complications.^[18]^ In our study, the incidence of lung infection was high. Patients with lung infection accounted for 82.35% (ERAS group) and 77.77% (traditional group) of the patients with postoperative complications. The incidence of other complications was similar to previous studies.

The incidence of postoperative lung and abdominal infection was significantly reduced in the ERAS group. Elderly patients with acute cholecystitis are prone to postoperative pulmonary infection due to advanced age, postoperative sputum resistance, tracheal intubation under general anesthesia, cross-infection, and long-term bed rest.^[19]^ There are few reports on the mechanism of pulmonary infection among elderly patients with acute cholecystitis. The potential reasons include the following: the pulmonary function of elderly patients progressively decreases; some elderly patients even have basic lung diseases, and surgical trauma will have a more pronounced adverse impact on lung function; elderly patients suffer from weakened respiratory muscle strength, weakened cough response, or are afraid to cough due to pain, leading to a decline in the ability of the body to actively expel bacteria; and acute cholecystitis requires longer anesthesia and surgery time, increasing the risk of infection. Pulmonary infections are commonly observed 48 hours post-cholecystectomy. Application of the ERAS protocol allows patients early ambulation and reduces pain, helps to expel sputum, and prevents lung infection. Previous evidence indicates that good intraoperative temperature control and avoiding hypothermia can reduce the incidence of postoperative infection, reduce intraoperative blood loss, and shorten postoperative anesthesia recovery time.^[20]^ Simultaneously, intraoperative monitoring of central venous pressure and intraoperative fluid infusion volume management can control circulating blood volume, reduce cardiac load, and avoid postoperative cardiac complications.

Biliary leakage is also a major concern for surgeons. Biliary leakage can result from injury to hepatocystic ducts or subvesical ducts when surgeons separate the gallbladder.^[21]^ Biliary leakage can also occur from slipping of the clips or faulty clip application. Blunt dissection in LC helps avoid biliary injury. Locking absorbable clips have also been shown to reduce the incidence of biliary leakage.^[22]^ To avoid biliary leakage, we chose to operate cautiously based on the perusing of preoperative imaging data, use blunt instruments to separate the tissue, and use Hem-o-lock clips after clearly identifying the gallbladder duct. In fact, there was no significant difference in the incidence of bile leakage between the 2 groups. ERAS protocol neither reduces the occurrence of biliary leakage nor causes more harm. Surgeons should be highly attentive during surgery to avoid biliary leakage.

There were 2 cases of urinary tract infection in the ERAS group and 4 cases in the traditional group, with no statistically significant difference. No indwelling of the catheter in ERAS did not significantly reduce the incidence of urinary tract infections, but neither did it increase the risk of urinary tract infections. No significant difference was observed regarding the incidences of biliary leakage and incision infection. Laparoscopic surgery itself can significantly reduce the risk of incision infection,^[23]^ so the ERAS protocol is not significantly effective.

The study had several limitations. First, based on the age range, the study sample was small. A multi-center study would shed more light on this question. Second, because the study was on the application of ERAS protocol in emergency surgery, some protocols were not implemented or were different from the conventional protocols. This could potentially make the ERAS protocol less effective. Third, the study was a retrospective study, and the cases in the ERAS group were mostly late, which may have an impact on postoperative outcomes, considering the surgery proficiency. Simultaneously, with the development of aging research, some associations have upgraded the definition of old age to >70 years old, and further study is necessary under this classification.

## 5. Conclusion

The application of the ERAS protocol in the perioperative management of elderly patients with acute cholecystitis was proven to promote rapid recovery, reduce postoperative complication incidence, and shorten hospital stay duration. The results of this study indicate that the application of ERAS protocol in perioperative management of LC is effective and beneficial for postoperative rehabilitation of patients.

## Acknowledgments

We would like to thank Editage (www.editage.cn) for English language editing.

## Author contributions

**Conceptualization:** Tianyang Yu, Luwen Zhao, Aijun Yu.

**Data curation:** Tianyang Yu, Hongtao Zhao, Hua Fu, Jian Li.

**Formal analysis:** Tianyang Yu, Luwen Zhao, Aijun Yu.

**Funding acquisition:** Aijun Yu.

**Investigation:** Tianyang Yu, Luwen Zhao, Hongtao Zhao, Hua Fu, Jian Li.

**Methodology:** Tianyang Yu, Aijun Yu.

**Project administration:** Tianyang Yu, Luwen Zhao, Aijun Yu.

**Supervision:** Tianyang Yu, Aijun Yu.

**Writing – original draft:** Tianyang Yu, Aijun Yu.

**Writing – review & editing:** Tianyang Yu, Luwen Zhao, Hongtao Zhao, Hua Fu, Jian Li, Aijun Yu.
